# Investigation of the understanding on tropical infectious diseases and oral health among dental professionals in China

**DOI:** 10.1186/s12903-022-02250-x

**Published:** 2022-06-02

**Authors:** Yi Shuai, Wenyin Zhu, Bingyao Liu, Ping Li, Lei Jin

**Affiliations:** 1 Department of Stomatology, Jinling Hospital, Medical School of Nanjing University, Nanjing, 210002 Jiangsu People’s Republic of China; 2 Department of Stomatology, General Hospital of Eastern Theater Command, PLA, Nanjing, 210002 Jiangsu People’s Republic of China; 3 Department of The Third Outpatient, Nanjing Stomatological Hospital, Medical School of Nanjing University, Nanjing, 210002 Jiangsu People’s Republic of China; 4 Institute of Liver Disease, Qinhuai Medical District of Jinling Hospital, Medical School of Nanjing University, Nanjing, 210002 Jiangsu People’s Republic of China

**Keywords:** Tropical infectious diseases, Oral health, Dental professionals, Clinical management, Knowledge

## Abstract

**Background:**

With the increasing exchange of domestic and international personnel, local infections of tropical infectious diseases are continuing in tropics and imported infections are emerging in non-tropics, some of which are accompanied by oral manifestations. Therefore, it is essential for dental professionals to identify the related oral manifestations, who are working for domestic medical service, international medical assistance, peace-keeping medical service or medical support of international joint military exercises. This study aims to investigate the attitude and knowledge of Chinese dental professionals on tropical infectious diseases and oral health, and to explore the difference between different genders, education backgrounds, professional identities, professional titles and tropics working experience.

**Methods:**

Network questionnaire was used to evaluate the knowledge and attitude of 236 Chinese dental professionals towards tropical infectious diseases and oral health.

**Results:**

The scores of the participants on tropical infectious diseases and oral health were quite low. Although working experience in the tropics partially affected the understanding, there was no difference between different genders, education backgrounds, professional identities and professional titles.

**Conclusion:**

The understanding of dental professionals on tropical infectious diseases and oral health was insufficient. It is necessary to improve the clinical education and management specified with tropical infectious diseases and oral health.

**Supplementary Information:**

The online version contains supplementary material available at 10.1186/s12903-022-02250-x.

## Background

Nowadays, personnel exchange has been largely increased because of increasingly open domestic and international cooperation, such as tourism, business, international assistance, peace-keeping action and international joint military exercises. As a result, sporadic and local epidemics of imported infectious diseases, being prevalent in tropics, have been reported in non-tropics [1–5]. Furthermore, the medical professionals might fight against tropical infectious diseases during their medical task of international assistance, peace-keeping action and international joint military exercises.

Oral-maxillofacial system has been reported to be affected by such infectious diseases, such as parasitic diseases [6, 7], viral diseases [8, 9], bacterial diseases [10, 11] and fungal diseases [10, 12]. In addition, oral adverse events of medications for tropical infectious diseases have been also reported [13]. Notably, some related oral manifestations occurred as the first sign of aforementioned infectious diseases [6–8, 14–16] and their drug associated adverse events [13]. Therefore, the dental professionals working for domestic medical service, international medical assistance, peace-keeping medical service or medical support of international joint military exercises might face a big challenge of managing tropical infectious diseases and oral health. Dental professionals should pay attention to identify the oral symptoms and infectious diseases, because some sufferers may firstly visit the dentists for their oral discomfort. Timely identification and appropriate management are particularly crucial for the management of infectious diseases in dental clinic, which largely depend on education and mastery of related knowledge. However, there are few studies concentrating on infectious diseases and oral health.

Therefore, the aim of current study is to uncover the attitude and knowledge of dental professionals on tropical infectious diseases and oral health, and to explore the difference between different genders, education backgrounds, professional identities, professional titles and tropics working experience. We expect that these findings will alert tropical doctors to improve clinical education and management of tropical medicine and oral health.

## Methods

### Questionnaire preparation

The network questionnaire (The Tencent Technology (Shenzhen) Co. Ltd, China), having been validated by a pilot study, was designed by three experts majoring in stomatology, infectious diseases and tropical medicine, respectively. Some changes were made based on the feedbacks of the pilot participants (21 participants from Department of Stomatology, Jinling Hospital, Medical School of Nanjing University), including linguistic errors and improper statements. The reliability and validity of the questionnaire were evaluated by Cronbach’s α Coefficient (0.92) and Goodness of Fit Index (0.83). The final questionnaire consisted of 56 questions, concerning general information (6 items), attitude towards tropical infectious diseases and oral health (5 items), understanding of tropical infectious diseases (24 items), tropical infectious diseases related oral health (17 items) and management of tropical infectious diseases in dental clinic (4 items).

### Study participants and analysis

By 2021, there were 278,000 registered dental professionals in China [17]. According to Doctor Law of The People's Republic of China, being registered in dental society is mandatory to practice in China. Totally, 241 dental professionals with practicing qualifications from 25 provincial regions of China were recruited in the study. Among them, 236 dental professionals completed the questionnaires. The proportions of the respondent backgrounds were close to the previous report, which covered all dental professionals from seven economically developed and underdeveloped provinces of different regions in China [18]. The participants of the pilot study were excluded from the main study. Dental professionals in the main study were recruited from multiple provincial regions through delivering announcement in the online chat room of Dental Professional Society of China. The recruited participants were asked for joining in a new online chat room to complete the network questionnaire. The questionnaires were distributed on Feb 22nd, 2020, and were all responded on Feb 27th, 2020. All the questionnaires were valid.

The data were divided into two parts: attitude and knowledge on tropical infectious diseases and oral health. The data were statistically analyzed between different genders, education backgrounds, professional identities, professional titles and tropics working experience. The score of each part of knowledge was normalized to 10, thus the total score was 80.

### Statistical methods

Descriptive statistics (number and percentage) was used to analyze general information of samples and attitude of the dental professionals towards tropical infectious diseases and oral health. Chi-square test or continuity correction was used to analyze the difference of ratio in different groups. Student’s t test and One-way ANOVA were used to analyze the Mean between different groups. The statistical significance was *p* < 0.05.

## Results

### Sample characteristics

A total of 241 dental professionals from 25 provincial regions of China were recruited as participants. However, 236 participants completed the questionnaires (97.9%). There were 121 males and 115 females; 174 postgraduates, 54 graduates and 8 junior college graduates; 211 doctors and 25 nurses; 26 participants with senior titles, 126 participants with intermediate titles, and 84 participants with junior titles; 24 participants with tropics working experience, 212 participants without such experience. The average age was 35.7 years old (23–59 years old). (Additional file 1: Table S1).

### The attitude of dental professionals towards tropical infectious diseases and oral health

Oral manifestations related to tropical infectious diseases have been displayed in Additional file 1: Table S2. The ratio of positive attention and systematical study experience of dental professionals on tropical infectious diseases and oral health were only 36.9% and 25.4%, respectively (Table 1). The positive ratio of participants who thought it was necessary to systematically learn about infectious diseases and oral health if working in non-tropics was 79.7%, while the ratio was statistically increased to 96.2% if working in tropics (Table 1). The positive ratio of participants, thinking identification of oral lesions was helpful to the management of tropical infectious diseases, was 85.6% (Table 1).

**Table 1:** The attitude of the dental professionals towards tropical infectious diseases and oral health

Content of questionnaire	Yes	No	Positive rate	Negative rate
Focus on tropical infectious diseases and oral health	87	149	36.9%	63.1%
Study on tropical infectious diseases and oral health systematically	60	176	25.4%	74.6%
If working in non-tropics, it is necessary to learn about infectious diseases and oral health systematically.^§^	188	48	79.7%	20.3%
If working in tropics, it is necessary to learn about infectious diseases and oral health systematically.^#^	227	9	96.2%	3.8%
Identification of oral lesions is helpful to the management of tropical infectious diseases	202	34	85.6%	14.4%

Item “#” compared with item “§”, *p* < 0.05

Compared to the participants with no tropics working experience, the participants with such experience showed statistically higher positive ratio on attention (58.3% vs 34.4%) and systematical study experience (41.7% vs 21.7%) of the dental professionals on tropical infectious diseases and oral health (Table 2). However, there was no difference between different genders, education backgrounds, professional identities and professional titles (Table 2).

**Table 2:** The attitude of the dental professionals towards tropical infectious diseases and oral health (classification)

Content of questionnaire	Gender			Education background				Professional identity			Professional title				Tropics working experience		
	Male (121)	Female (115)	*p value*	Post-graduate (174)	Graduate (54)	Junior (8)	*p value*	Doctor (211)	Nurse (25)	*p value*	Senior (26)	Intermediate (126)	Junior (84)	*p value*	Yes (24)	No (212)	*p value*
	Yes/No	Yes/No		Yes/No	Yes/No	Yes/No		Yes/No	Yes/No		Yes/No	Yes/No	Yes/No		Yes/No	Yes/No	
Focus on tropical infectious diseases and oral health	51/70 42.1%	36/79 31.3%	0.08	63/111 36.2%	22/32 40.7%	2/6 25.0%	0.65^△^	78/13335.3%	9/16 36.0%	0.98	11/15 42.3%	44/82 34.9%	32/52 38.1%	0.74^△^	14/10 58.3%	73/13934.4%	0.02*
Study on tropical infectious diseases and oral health systematically	34/87 28.1%	22/93 19.1%	0.11	44/130 25.3%	12/42 22.2%	0/8 0%	0.25^△^	49/162 22.2%	7/18 28.0%	0.82	7/19 26.9%	32/94 25.4%	17/67 20.2%	0.64^△^	10/14 41.7%	46/166 21.7%	0.03*
If working in non-tropics, it is necessary to learn about infectious diseases and oral health systematically. ^§^	97/24 80.2%	94/21 81.7%	0.76	135/39 77.6%	48/6 88.9%	6/2 75.0%	0.07^△^	169/42 76.5%	22/3 88.0%	0.44	23/3 88.5%	101/25 80.2%	67/17 79.8%	0.58^△^	23/1 95.8%	168/44 79.2%	0.09
If working in tropics, it is necessary to learn about infectious diseases and oral health systematically. ^#^	117/4 96.7%	110/5 95.7%	0.68	168/6 96.6%	52/2 96.3%	7/1 87.5%	0.43^△^	204/7 92.3%	23/2 92.0%	0.26	25/1 96.2%	120/6 95.2%	82/2 97.6%	0.67^△^	24/0 100%	203/9 95.8%	0.64
Identification of oral lesions is helpful to the management of tropical infectious diseases	106/15 87.6%	96/19 83.5%	0.37	149/25 85.6%	47/7 87.0%	6/2 75.0%	0.66^△^	179/32 81.0%	23/2 92.0%	0.30	23/3 88.5%	106/20 84.1%	73/11 86.9%	0.26^△^	21/3 87.5%	181/31 85.4%	0.37

Item “*”, *p* < 0.05. Item “^△^”: *p* value of three sub-groups comparison, there was no difference between any two sub-groups. Item “#” compared with item “§”, *p* < 0.05

### The understanding of dental professionals on tropical infectious diseases and oral health

**Fig. 1:**
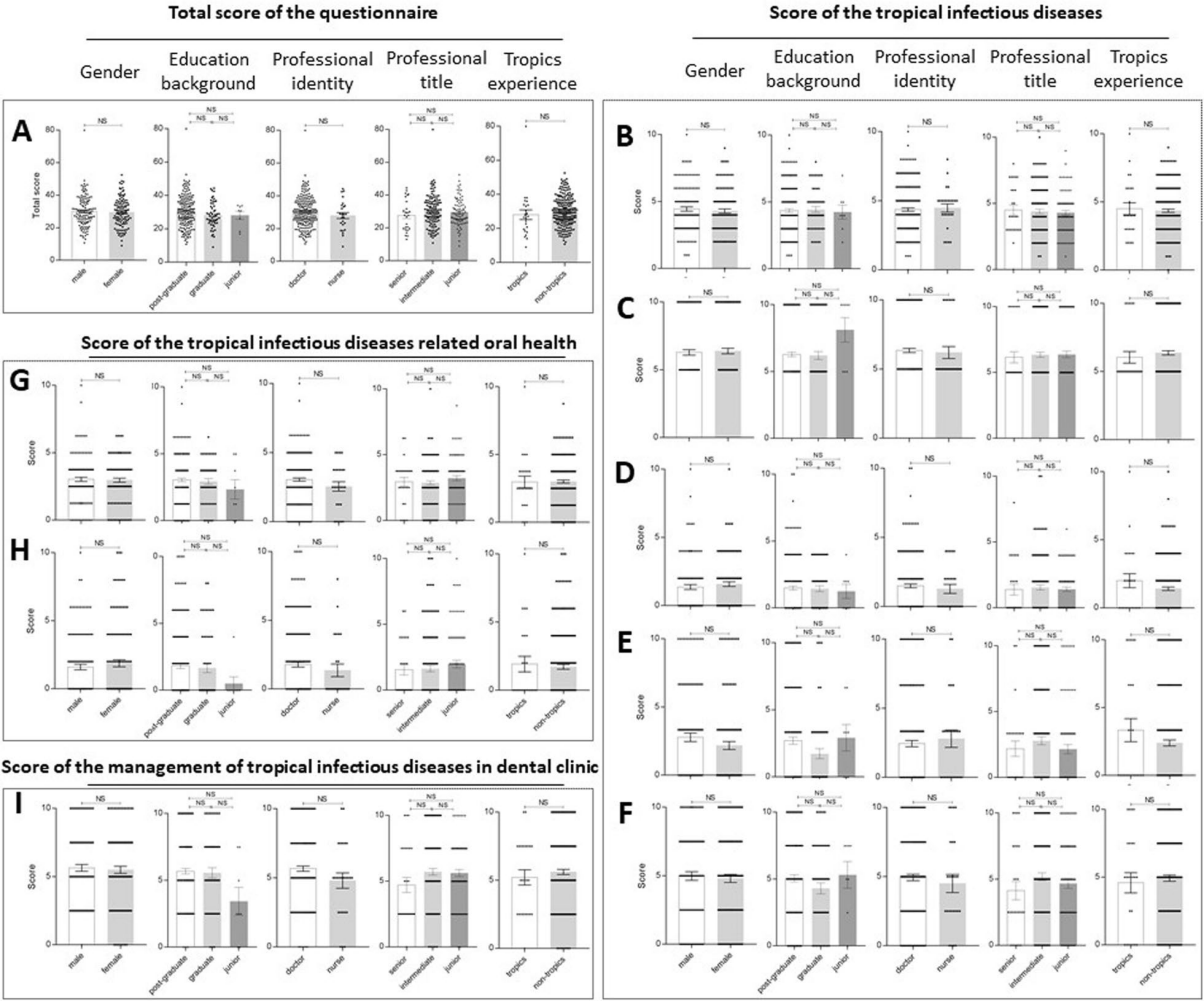
Score of the questionnaire. Comparison of Total score of the questionnaire (**A**); Score of the tropical infectious diseases (Scope (**B**), Subtype (**C**), Transmission route (**D**), Insect-borne type (**E**) and Associated pathogen (**F**) of tropical infectious diseases); Score of the tropical infectious diseases related oral health (Related oral manifestations (**G**), Oral adverse events of medications for tropical infectious diseases (**H**)); Score of the management of tropical infectious diseases in dental clinic (**I**) between different genders, education backgrounds, professional identities, professional titles and tropics working experiences

The average of total score was only 29.87 ± 0.60 (Fig. 1A). In the section of tropical infectious diseases, the average score of scope (Fig. 1B), prevalent subtype (Fig. 1C), transmission route (Fig. 1D), insect-borne type (Fig. 1E), associated pathogen (Fig. 1F) of tropical infectious diseases were 4.37 ± 0.11, 6.33 ± 0.14, 1.48 ± 0.12, 2.49 ± 0.21 and 4.89 ± 0.22, respectively. In the section of tropical infectious diseases related oral health, the average score of oral manifestations of tropical infectious diseases (Fig. 1G), oral adverse events of medications for tropical infectious diseases (Fig. 1H) were 2.99 ± 0.11, 1.74 ± 0.17, respectively. In the section of management of tropical infectious diseases in dental clinic, the average score was 5.57 ± 0.18 (Fig. 1I).

However, there was no difference of score between different genders, education backgrounds, professional identities and professional titles and tropics working experiences in aforementioned sections (Fig. 1).

## Discussion

Dental clinics are at high risk of tropical infectious diseases because: (1) some infected patients or patients with drug-induced adverse events, need to be identified, may firstly visit the dentists for their accompanied oral discomfort [6–8, 13–16]; (2) the aerosols will be generated during the oral treatment, which is the crucial risk factor for infectious disease spread [19–21]; (3) the closed treatment environment is easy for infectious disease spread [19, 20]; (4) the blood exposure is another risk factor for infectious disease spread[22]; (5) some tropical infectious diseases have severe late-onset oral complications which need follow-up, such as Burkitt lymphoma related to malaria [7], oral cancer related to trichinosis [6] and jawbone osteonecrosis related to dengue fever [8]. Therefore, it is necessary for the dental professionals to focus on tropical infectious diseases and oral health. The understanding of dental professionals on this topic will directly influence the clinical management of infectious diseases in dental clinics.

The network questionnaire was formulated to evaluate the understanding of the dental professionals on tropical infectious diseases and oral health: (1) The attention and study experience of the dental professionals on tropical infectious diseases was insufficient. Tropics working experience positively improved the attention and study experience of the dental professionals on this topic, whereas gender, education backgrounds, professional identities and professional titles did not affect it. It is indicated that the participants with tropics working experience might have seen some suspected cases during their clinical practice to improve the attention and study experience. (2) Most dental professionals realized the importance of study on tropical infectious diseases and oral health. Additionally, most dental professionals realized that it was helpful for the management of tropical infectious diseases through oral manifestation identification. All these suggest that the dental professionals’ attitude was positive on this topic. (3) The low score of the tropical infectious diseases and oral health suggested that mastery of the knowledge was poor among the dental professionals. In addition, gender, education backgrounds, professional identities, professional titles and tropics working experience could not ameliorate the understanding of knowledge, suggesting that (1) academic education, continuing education and clinical education are not satisfied on this topic; (2) some suspected cases might be misdiagnosed by some dental professionals; (3) learning and mastery of the knowledge was insufficient among the participants with tropics working experiences, although they have seen some suspected cases and realized the importance of tropical infectious diseases and oral health.

With regard to the tropical infectious diseases and oral health, there were three types of surveys exploring knowledge mastery of medical and dental providers. Firstly, most research reported the knowledge and attitude of medical or dental professionals towards infectious diseases, such as AIDS [23, 24], hepatitis [25, 26], COVID [27, 28], leprosy [29, 30] and some other tropical diseases [31, 32]. However, few of them concentrated on their oral manifestations. Additionally, infectious diseases involved in most of these surveys were not tropical infectious diseases. Secondly, several studies investigated the understanding of the medical and dental professionals on oral signs related to systemic diseases [33], whereas tropical infectious diseases were always ignored. Thirdly, although some oral disorders related to tropical infectious diseases have been found in clinic [8, 10, 11], the researchers did not investigate the understanding of this knowledge and importance among dental professionals. However, our study paid attention to such neglected aspect of tropical infectious diseases and oral health. In addition, most of the previous surveys included only one or two classes of medical or dental professionals, therefore the outcomes might not be representative of the overall professionals.

Lack of the knowledge and understanding of this topic might bring certain risks for the management of infectious diseases in dental clinics, such as misdiagnosis, missed diagnosis, delayed diagnosis and even leading to spread of infectious diseases. Therefore, it is urgent to develop an educational and training system for tropical infectious diseases and oral health: (1) Compile textbooks on tropical infectious diseases and oral health, including the knowledge about tropical infectious diseases; associated oral manifestations; oral adverse events of commonly used drugs for infectious diseases; clinical management; emergency treatment and follow-up treatment instructions, and so forth. (2) Offer public course of this topic in academic education, continuing education and clinical education. (3) Offer targeted guidance and training for the dental professionals who will be working in tropics, such as international medical assistance, peace-keeping medical service and medical support of international joint military exercises. (4) Establish a public communication platform to share popular scientific information, case report and discussion of this topic. We hope to raise concerns of this topic and improve the role of the dental professionals in the management of tropical infectious diseases through aforementioned measures.

## Conclusion

Tropical infectious diseases are highly related to oral health, neglect of which might trigger the spread of infectious diseases. We first investigated the understanding of tropical infectious diseases and oral health among dental professionals, which was neglected by most medical professionals and researchers. The data of the investigation showed that the dental professionals were lacking in understanding on tropical infectious diseases and oral health, which might bring hidden danger to oral and public health. It is necessary to improve dental education and management specified with infectious diseases and oral health.

## Supplementary Information

**Additional file 1:** Sample characteristics of the study.

## Data Availability

All data generated or analyzed during this study are included in this published article and its Additional files.
