# Supplementary material for: Investigation of the understanding on tropical infectious diseases and oral health among dental professionals in China
Source: BMC Oral Health. 2022 Jun 2;22:219. doi: 10.1186/s12903-022-02250-x (PMC9164551; doi:10.1186/s12903-022-02250-x)
Supplement: Supplementary file 1 — Additional file 1: Sample characteristics of the study. [file 12903_2022_2250_MOESM1_ESM.docx]

**Supplementary materials**

| Table S1. Sample characteristics. | |
| --- | --- |
| Category | Composition |
| Sample size | 236 |
| Gender | Male (121), Female (115) |
| Age | Average 35.7 Y (23Y-59Y) |
| Education background | Postgraduate (174), Graduate (54), Junior (8) |
| Professional identity | Doctor (221), Nurse (25) |
| Professional title | Senior (26), Intermediate (126), Junior (86) |
| Tropics working experience | Yes (24), No (212) |

| Table S2. Oral manifestations related to tropical infectious diseases | | |
| --- | --- | --- |
| Category | | Related oral manifestations |
| Parasitic diseases[1-7] | | |
| Malaria | Gingival bleeding; oral ulcer; oral pigment; glossitis; pericoronitis; alveolar bone resorption; herpes labialis; herpes gingivostomatitis; burkitt lymphoma of jaw; enamel hypoplasias; bitter taste; sore throat | |
| Amoebasis | Moist tongue; furred tongue | |
| Leishmaniasis | Painful, destructive granulomatous lesions of facial skin, lips, buccal mucosa, palate and tongue | |
| Trypanosomiasis | Lipochagomata genii (painful, ovoid, purple patches of buccal bilateral fat pad); myoclonus of lips | |
| Ancylostomiasis | Extreme pallor of oral mucosa and lips; glossodynia; atrophy of lingual papilla; angular cheilosis | |
| Trichuriasis | Ulcerative stomatitis; glossitis; hyperplastic gingivitis | |
| Filariasis | Asymptomatic, solitary swelling of lips, tongue, gingival papillae and buccal mucosa; oral manifestations mainly occur in female sufferers | |
| Trichinosis | Recurrent mandibular swelling with pain; diffuse indefinite radiolucency on the alveolar crest; hyperplastic gingivitis; dry mouth; oral ulceration; facial myalgia; difficulty in mastication, deglutition and speech; associated with oral squamous cell carcinoma | |
| Taeniasis | Ulcerative, hypertrophic or hemorrhagic stomatitis, gingivitis and stomadynia | |
| Cysticercosis | Cystic nodules on the tongue, buccal mucosa, lips and facial skin | |
| Echinococcosis | Painless, solitary, firm or fluctuant swelling of hydatid cysts in major salivary glands, jaw bones, tongue and buccal mucosa | |
| Sparganosis | Asymptomatic submandibular or labial mass | |
| Bacterial diseases[8, 9] | | |
| Yaws | Gangosa (destruction of hard palate and maxillary nasal processes with heavy scarring) | |
| Leprosy | Hemorrhagic sessile nodules, ulceration, necrosis and fibrosis of oral mucosa; lepromata on the tongue; fissured tongue; atrophic papillae and loss of taste; smokers palate; oral melanosis; oral depigmentation; oral candidiasis; gingivitis; periodontitis; loosening, dysplasia and dental pulp necrosis of maxillary anterior teeth; erythematous infiltrated plaque of facial part | |
| Viral diseases[10-12] | | |
| Dengue fever | Acute hemorrhage of gingiva, palate and tongue; post-extraction hemorrhage; blisters of tongue and palate; erythematous plaque of oral mucosa; dry mouth; taste changes; osteonecrosis of the jaw; oral candidiasis; swallowing difficulty | |
| Measles | Facial rashes; Koplik's spots on oral mucosa, oral ulceration; gingivitis; pericoronitis; necrotic stomatitis; oral candidiasis | |
| Fungal diseases[8, 13-17] | | |
| Paracoccidioidomycosis | Painful proliferative erythematous granulomata of lips, tongue, gingiva, palate, alveolar ridge, pharynx, labial and buccal mucosa with ulceration, gingival hemorrhage; loosening of teeth; pigmentation of oral mucosa; fibrosis and cicatrization; facial lymphadenopathy; sialorrhea | |
| Histoplasmosis | Granulomas or fungating ulcers on lips, tongue, buccal mucosa, palate, gingiva and alveolar ridge with an indurated border; sore pharynx and larynx; dysphonia; dysphagia | |
| Rhinosporidiosis | Exuberant granulomatous lesions of jawbone, tongue, soft palate or oropharynx | |
| Oral adverse events related to medications for tropical infectious diseases[18] | | |
| Antimalarials | Oral soft tissue pain; toothache; oral paresthesia; enamel hypoplasia; tooth discoloration; oral ulceration; periodontal diseases; oral hemorrhage; stomatitis; simple cutaneous and mucosal lesions; facial herpes zoster; herpes labialis; oral lichenoid reaction; oral hyperpigmentation; tongue disorders or symptoms; salivary gland disorders; tonsillitis; bitter taste; halitosis; facial swelling; facial skin lesions of severe system adverse events; sore throat | |

1. Hassona Y, Scully C, Delgado-Azanero W, de Almeida OP: **Oral helminthic infestations**. *Journal of investigative and clinical dentistry* 2015, **6**(2):99-107.

2. Zislis T, Adrian JC, Cutright DE: **Oral manifestations of tropical infectious diseases of Central and South America. Part I: Protozoan and helminthic infections**. *Military medicine* 1980, **145**(8):525-528.

3. Shuai Y, Liu B, Zhou G, Rong L, Niu C, Jin L: **Oral manifestations related to malaria: A systematic review**. *Oral Dis* 2021, **27**(7):1616-1620.

4. Mignogna MD, Celentano A, Leuci S, Cascone M, Adamo D, Ruoppo E, Favia G: **Mucosal leishmaniasis with primary oral involvement: a case series and a review of the literature**. *Oral Dis* 2015, **21**(1):e70-78.

5. Frost L: **Dental management of the tropical disease human African trypanosomiasis: an unusual case of pseudobulbar palsy**. *British dental journal* 2011, **210**(1):13-16.

6. Hertzman PA, Blevins WL, Mayer J, Greenfield B, Ting M, Gleich GJ: **Association of the eosinophilia-myalgia syndrome with the ingestion of tryptophan**. *N Engl J Med* 1990, **322**(13):869-873.

7. Ivanov KS, Antonov VS, Knysh GG, Khadzhaeva AN, Antykova LP, Lavrova VP, Nepomniashchaia LP, Kudriavtseva OV, Aleksandrova LI: **[The clinical characteristics of 2 outbreaks of trichinelliasis]**. *Med Parazitol (Mosk)* 1990(4):41-42.

8. Zislis T, Adrian JC, Cutright DE: **Oral manifestations of tropical infectious diseases of Central and South America. Part II: Bacterial and mycotic infections**. *Military medicine* 1980, **145**(8):529-534.

9. Vohra P, Rahman MSU, Subhada B, Tiwari RVC, Nabeel Althaf MS, Gahlawat M: **Oral manifestation in leprosy: A cross-sectional study of 100 cases with literature review**. *Journal of family medicine and primary care* 2019, **8**(11):3689-3694.

10. Pedrosa MS, de Paiva M, Oliveira L, Pereira S, da Silva C, Pompeu J: **Oral manifestations related to dengue fever: a systematic review of the literature**. *Australian dental journal* 2017, **62**(4):404-411.

11. Katz J, Guelmann M, Stavropolous F, Heft M: **Gingival and other oral manifestations in measles virus infection**. *J Clin Periodontol* 2003, **30**(7):665-668.

12. Neville BW, Damm DD, Allen CM, Bouquot JE: **Oral and maxillofacial pathology, 2nd edition**. *Philadelphia: Saunders* 2002:231.

13. Souza RL, Bonan PR, Pinto MB, Prado JD, de Castro JF, Carvalho EA, Perez DC: **Oral paracoccidioidomycosis in a non-endemic region from Brazil: A short case series**. *Journal of clinical and experimental dentistry* 2019, **11**(10):e865-e870.

14. Ferreira OG, Cardoso SV, Borges AS, Ferreira MS, Loyola AM: **Oral histoplasmosis in Brazil**. *Oral Surg Oral Med Oral Pathol Oral Radiol Endod* 2002, **93**(6):654-659.

15. Balaji SM, Balaji P: **Maxillary rehabilitation after complete destruction by fungal osteomyelitis**. *Indian journal of dental research : official publication of Indian Society for Dental Research* 2019, **30**(5):807-809.

16. Rath R, Baig SA, Debata T: **Rhinosporidiosis presenting as an oropharyngeal mass: A clinical predicament?** *Journal of natural science, biology, and medicine* 2015, **6**(1):241-245.

17. Prasad K, Veena S, Permi HS, Teerthanath S, Shetty KP, Shetty JP: **Disseminated cutaneous rhinosporidiosis**. *Journal of laboratory physicians* 2010, **2**(1):44-46.

18. Shuai Y, Wang J, Jiang H, Yu Y, Jin L: **Oral-maxillofacial adverse events related to antimalarials**. *Oral Dis* 2020.

**References**
